# Central Dentinogenic Ghost Cell Tumor: A Rare Entity

**DOI:** 10.7759/cureus.78556

**Published:** 2025-02-05

**Authors:** Mary Nashashibi, Amir Farah, Imad Abu El-Naaj, Munir Nashashibi

**Affiliations:** 1 Pathology, Tzafon Medical Center, Azrieli Faculty of Medicine, Bar Ilan University, Tiberias, ISR; 2 Surgery, Medical College of Wisconsin, Milwaukee, USA; 3 Oral and Maxillofacial Surgery, Tzafon Medical Center, Azrieli Faculty of Medicine, Bar Ilan University, Tiberias, ISR

**Keywords:** central dentinogenic ghost cell tumor, dgct, gcoc, ghost cell odontogenic carcinoma, oncology, pathology, surgery

## Abstract

Odontogenic tumors are rare lesions with varied clinical presentations and behaviors. A dentinogenic ghost cell tumor (DGCT) is a rare odontogenic neoplasm that can be classified into central (intraosseous) and peripheral (extraosseous) types, with a central DGCT often presenting as a bone-expanding lesion. We report a case of a multimorbid 66-year-old male patient with a central DGCT presenting as a painless, 3 × 5.3 cm expansile mass in the right mandibular body. The patient underwent radiographic and histologic assessment followed by tumor enucleation under local anesthesia, which revealed classic features of the DGCT, including ghost cells, calcifications, and a low proliferation index, consistent with low malignant potential.

## Introduction

An odontogenic tumor is a rare neoplasm originating from the tissues involved in tooth development, including epithelial, mesenchymal, or combined components, with behavior ranging from benign and slow-growing to aggressive and potentially malignant [1]. The incidence of odontogenic tumors varies greatly by region, constituting 1% of oral pathologies in North America compared to up to 19% in certain African countries [2].

A dentinogenic ghost cell tumor (DGCT) and ghost cell odontogenic carcinoma (GCOC) are rare odontogenic neoplasms representing the benign and malignant ends of the same pathological spectrum [3]. A DGCT can manifest as either an intraosseous lesion (central DGCT (DGCTc)) located within the jawbone, primarily in the first molar to canine region, or as an extraosseous peripheral lesion (DGCTp) arising in the gingiva or alveolar mucosa [4,5]. DGCTs and GCOC share significant clinicopathological similarities, necessitating meticulous histopathological evaluation to differentiate them, particularly due to the potential for malignant transformation of DGCTs [3].

However, the rarity of this entity, along with heterogeneous reports on categorization criteria and treatment decisions, often leads to reliance on small case series that frequently lack complete radiographic or histological data. Here, we present a rare case of a 66-year-old multimorbid man diagnosed with a DGCTc, who presented with a large right mandibular odontogenic tumor, comprehensively detailing its clinical, radiographic, and pathological diagnostic features.

## Case presentation

A 66-year-old man with a complex medical history presented with multiple chronic conditions, including diabetes mellitus, hyperlipidemia, gout, hypertension, ischemic heart disease, and moderate-stage congestive heart failure. Past interventions included multiple cardiac catheterizations and coronary artery bypass graft surgery performed ten years ago. Additionally, he had a history of deep venous thrombosis, stage 4 chronic renal failure, epilepsy, and chronic obstructive pulmonary disease related to heavy smoking.

**Figure 1:**
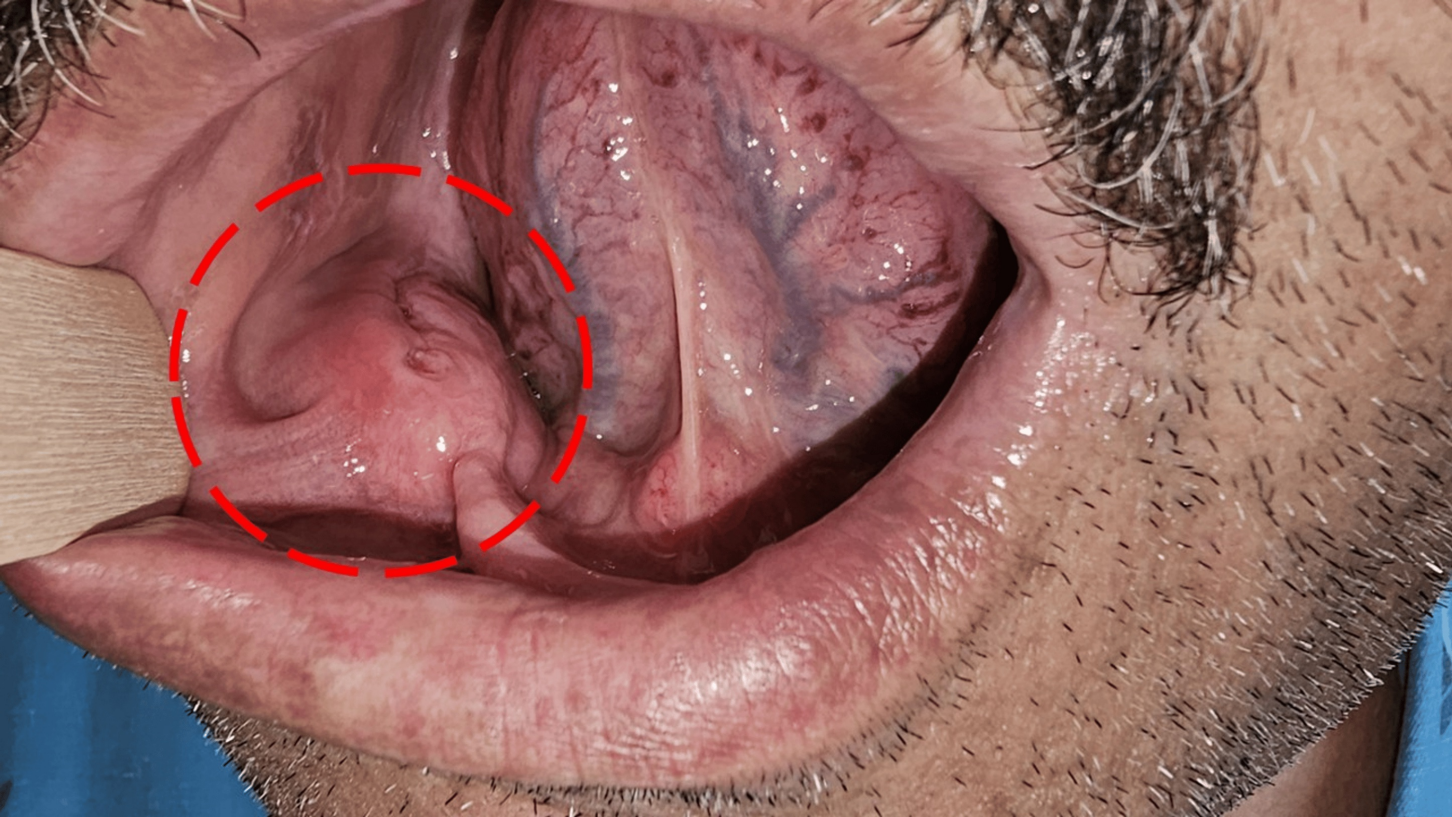
Clinical Presentation of a Right Posterior Alveolar Ridge Mass Soft, non-fluctuant mass originating from the right posterior alveolar ridge (red circle).

**Figure 2:**
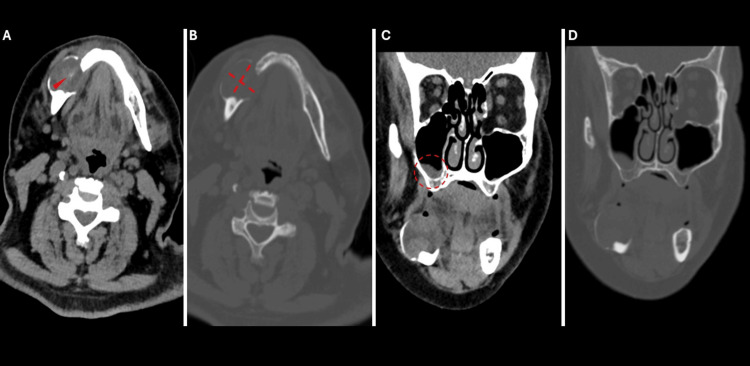
CT Imaging of the Right Mandibular Expansile Mass Non-contrast CT images of a rounded, heterogeneous, expansile mass in the right mandibular body measuring 3 x 5.3 cm: (a) Soft tissue window, transverse plane: The mass contains a small hyperdense focus, likely representing a blood vessel or hemorrhage. (b) Bone window, transverse plane: The mass demonstrates cortical and bony structure destruction. (c) Soft tissue window, coronal plane: The mass is in direct contact with the mylohyoid and platysma muscles. A retention cyst is observed in the right maxillary sinus, along with mucosal thickening in the left ethmoid cells. (d) Bone window, coronal plane: Additional visualization of the mass and its bony involvement.

The patient presented with a soft, non-fluctuant mass originating from the right posterior alveolar ridge (Figure 1). The mass was described as painless and non-tender. The remainder of the physical examination yielded no clinically significant findings. No functional impairment was reported. Subsequently, a non-contrast CT scan (due to renal failure) revealed a rounded, heterogeneous, expansile mass in the right mandibular body, measuring 30 x 53 mm, causing cortical and bony destruction. The mass was in direct contact with the mylohyoid and platysma muscles. Notably, a retention cyst was observed in the right maxillary sinus, with no enlarged lymph nodes in the neck or upper mediastinum (Figure 2).

An incisional biopsy was performed, revealing classic histopathological features of a DGCT, including islands of odontogenic epithelium resembling that of ameloblastoma, with minor cystic formations observed.

The initial treatment plan involved segmental resection of the mandible with reconstruction using a fibular free flap, a standard surgical procedure for replacing bone removed during cancer treatment, particularly in the upper or lower jaw. However, due to the patient's severe comorbidities and high anesthesia risk, the treatment approach was modified to full tumor enucleation via an intraoral approach. This decision was supported by the encapsulated nature of the tumor, which permitted uncomplicated removal while minimizing the risk of morbidity. Enucleation is a less invasive procedure, often requiring a shorter operative time and, in many cases, only local anesthesia, reducing overall patient risk. Following multidisciplinary team discussions, a consensus was reached to proceed with the enucleation using sub-periosteal dissection to ensure complete removal of the lesion while preserving the surrounding structures. Hemostasis was effectively achieved using diathermy, and the operative site was closed primarily.

Macroscopically, the lesion was encapsulated, tissue-based, bone-resorbing, and contained turbid fluid with irregular borders, measuring approximately 20 mm in diameter. Gross necrosis was not reported.

**Figure 3:**
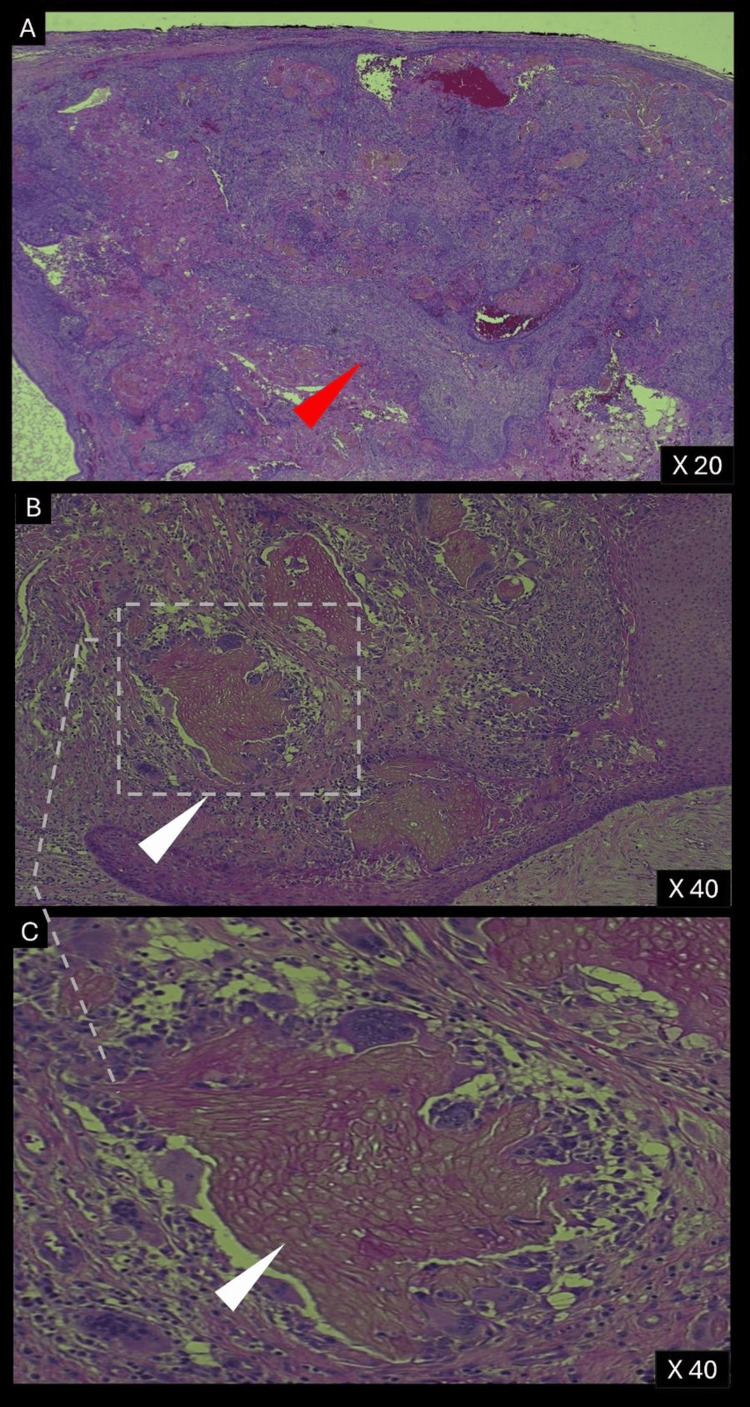
Histopathological Features of the Tumor Hematoxylin and eosin stain: (a) 20X magnification. (b and c) 40X magnification. The predominantly solid mass consists of sheets of anastomosing cords and strands of odontogenic epithelium (red arrow), microcystic development, and admixed ghost cells (anucleate epithelial cells with pale cytoplasm containing cytoplasmic clearings, representing the location of a previously resorbed nucleus or organelles) (white arrow). The mass also contains interspersed islands of cells with squamous differentiation, ameloblastic-like areas with palisading basaloid cells, and varying levels of dentinoid and cementum-like calcified collagenous matrix.

**Figure 4:**
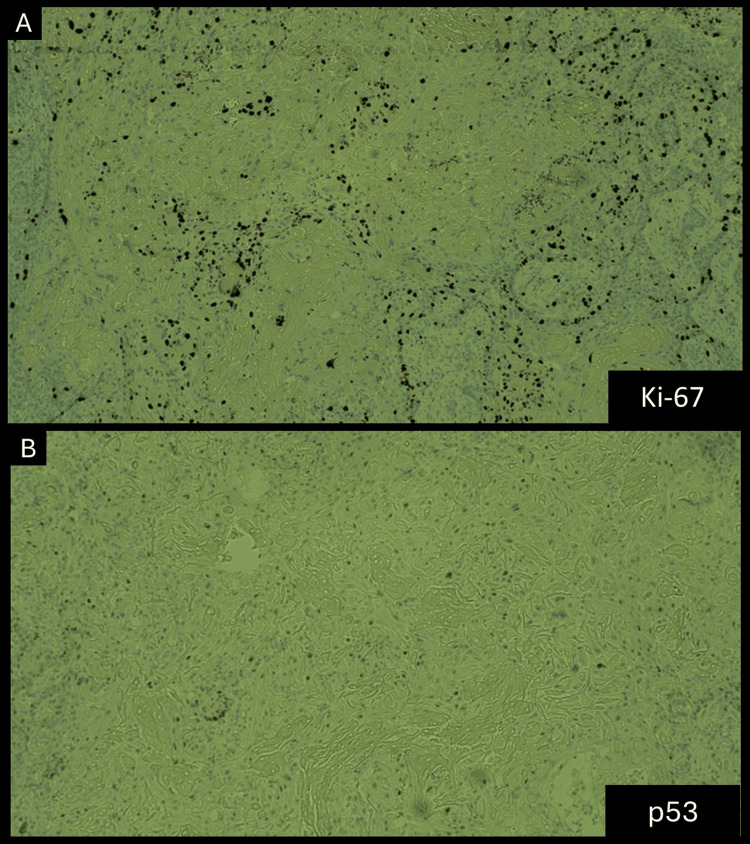
(A) Ki-67 staining. (B) p53 staining. (A) Low proliferation index as expressed by Ki-67 (<5%) and (B) Minimal expression of p53 immunohistochemical staining.

Microscopically, the lesion was lined by oral epithelium, and within the connective tissue stroma, an epithelial odontogenic tumor was identified (Figure 3). The tumor contained cystic structures lined by odontogenic epithelium with ameloblastic features, ghost cells, and calcifications. Numerous islands of tumor cells were observed within the cyst wall, surrounded by a multinucleated giant cell reaction directed at the ghost cells. Immunohistochemical studies showed a Ki-67 proliferation index of 3-5% (Figure 4A), indicating low malignant potential, while p53 expression was minimal (Figure 4B), and finally BRAF mutation was negative. These histopathological and immunohistochemical findings confirmed the diagnosis of a DGCT.

At his most recent follow-up, one month after the procedure, the physical examination revealed a well-healed wound. 

## Discussion

DGCTs and GCOC are rare odontogenic tumors representing the benign and malignant ends of a shared pathological spectrum. They account for approximately 5% and 2.5% of all ghost cell odontogenic lesions, respectively, and comprise less than 0.5% of all odontogenic tumors [6,7].

Over 80% of DGCTs present as intraosseous lesions (also known as central DGCTs or DGCTc), typically located in the region between the first molar and canine [4,5]. The remaining minority of cases present as extraosseous peripheral lesions, arising from the gingiva or alveolar mucosa [4,5]. DGCTp typically presents as a firm, painless nodular lesion on the oral mucosa, measuring up to 3.0 cm [3]. Clinically, it may resemble common reactive lesions of the oral mucosa. When imaging findings are present, a slight "cup-shaped" erosion of the underlying cortical bone is often observed [3]. In contrast, the DGCTc generally presents as a larger, expansile mass within the jawbone, often associated with significant bone destruction or expansion. Radiographically, central DGCTs are typically characterized by mixed unilocular or multilocular images which may lead to bone cortex expansion with well-defined margins [3].

Histologically, both peripheral and central DGCTs share similar features, including islands of odontogenic epithelial cells within a mature connective tissue resembling ameloblastoma, with occasional minor cyst formation within the epithelial islands [8].

However, central DGCTs are more likely to exhibit direct bone involvement, and they have a higher potential for malignant transformation compared to their peripheral counterparts, which are less likely to progress to malignancy [9]. Therefore, management strategies for central DGCTs may involve more extensive surgical procedures, such as bone resection, while peripheral DGCTs are typically treated with simpler excision of the soft tissue lesion.

In our case, the incisional biopsy showing benign features, the well-rounded shape of the tumor on the CT scan, the absence of lymphadenopathy, and the patient’s significant comorbidities, which influenced the decision-making process regarding the extent of the surgery, all supported localized surgical enucleation as a single treatment modality. Ideally, jaw reconstruction could have been performed if the patient had been amenable to general anesthesia for a more advanced surgical procedure. Clinical and radiographic follow-ups are planned, with the knowledge that high recurrence rates are of this entity’s characteristics [3].

The relationship between DGCTs and GCOC, the malignant counterpart on the spectrum, warrants emphasis (Table 1) [10-19]. The progression of DGCTs into GCOC represents a critical pathway in the malignancy's development. GCOC may also arise from a calcifying odontogenic cyst, the cystic variant within the spectrum of ghost cell lesions, or as a de novo malignant neoplasm [10]. Its potential for regional and distant metastasis underscores its aggressive behavior. More research is needed to establish a definitive link between DGCT and GCOC transformation.

**Table 1:** Comparison of Peripheral DGCTs, Central DGCTs, and GCOC. DGCT: Dentinogenic ghost cell tumor

Feature	Peripheral DGCT (DGCTp)	Central DGCT (DGCTc)	Ghost Cell Odontogenic Carcinoma (GCOC)
Definition	Extraosseous benign odontogenic tumor with limited growth potential.	Intraosseous benign odontogenic tumor with aggressive local behavior and recurrence risk.	Malignant odontogenic neoplasm, often with aggressive growth, recurrence, and potential for metastasis.
Prevalence	A minority of DGCT cases (~17%); less common than central DGCTs; DGCTs represent approximately 5% of all ghost cell odontogenic lesions and 0.38% of all odontogenic tumors [4,6,7].	Majority of DGCT cases (~83%); DGCTs represent approximately 5% of all ghost cell odontogenic lesions and 0.38% of all odontogenic tumors [4,6,7]	Represents approximately 2.5% of all ghost cell odontogenic lesions, and 0.23% of all odontogenic tumors [6,7].
Location	Extraosseous, commonly in gingiva or alveolar ridge mucosa; predilection for anterior mandible [4].	Jawbone (intraosseous), predominantly occurs in the first molar to canine region [5].	Most commonly found in the maxillary bone [17].
Age	Occurs later in life than the central type [4].	Occurs more frequently in younger individuals when compared to peripheral DGCTs [3].	Occurs more frequently in younger individuals when compared to peripheral DGCTs [3].
Size	A firm, painless nodular lesion on the mucosa, measuring up to 3.0 cm [3].	Larger expansile mass ranging in size from 1 cm to over 10 cm in diameter [18].	Ranging from 3 mm to 10 cm [19].
Clinical Features	Painless, slow-growing exophytic mass; often asymptomatic [4].	Swelling, bone expansion, and occasional pain; may cause tooth displacement or mobility [18].	Painful swelling often accompanied by local paresthesia [19].
Radiographic Features	Slight "cup-shaped" erosion of the underlying bone [3].	Mixed unilocular or multilocular images, commonly associated with well-demarcated margins and cortical bone expansion [3].	Mixed radiolucent and radiopaque features with indistinct edges, often accompanied by tooth displacement and root resorption [19].
Histopathology	The histopathological hallmark of both central and peripheral DGCTs is the presence of sheets and rounded islands of odontogenic epithelial cells in a mature connective tissue setting, with a resemblance of ameloblastoma, with no mitoses and infrequent cyst formation [8].	The histopathological hallmark of both central and peripheral DGCTs is the presence of sheets and rounded islands of odontogenic epithelial cells in a mature connective tissue setting, with a resemblance of ameloblastoma, with no mitoses and infrequent cyst formation [8].	Malignant cells with ghost cells, high mitotic activity, and pleomorphism [10].
Aggressiveness	Less aggressive [15,16].	Locally aggressive, recurrence rate of up to 71% [15].	Highly aggressive, Metastasis occurred in 16.7% of cases [3].
Management	Local excision is sufficient [9].	Requires enucleation or surgical resection which can be segmental resection or en bloc. Long-term follow-up mandatory due to high recurrence risk [15,16].	Wide surgical resection with no less than a 5 mm of clear margins; radiotherapy/chemotherapy may be used [10].
Proliferation Index (Ki-67)	Low (<5%), indicating low malignant potential.	Low (3–5%), higher than peripheral DGCTs but lower than GCOC [12,13].	High, indicating aggressive behavior [12,13].
Recurrence	Recurrence is rare [15,16].	High recurrence rates [3].	High recurrence rate; five-year survival rate is ~73% [3].

DGCTs and GCOC have several overlapping clinicopathological characteristics. Both DGCTs and GCOC exhibit a male predominance, with DGCTs demonstrating a predilection for the mandible and GCOC for the maxilla. Peripheral DGCTs predominantly affect elderly individuals, whereas central DGCTs and GCOC are more commonly observed in younger patients [3].

Imaging proved valuable for differentiation, with malignant cases often showing cortical bone destruction and ill-defined margins, while benign lesions were typically well-demarcated and caused cortical expansion. However, exceptions to these patterns were noted [3]. DGCTs and GCOC are diagnosed microscopically by examining the proliferative odontogenic epithelium for cytological atypia and ghost cells, which may be accompanied by the dentinoid material [1,11].

According to the WHO, DGCTs predominantly exhibit an ameloblastomatous pattern with less prominent basaloid epithelium. In contrast, GCOC consists of malignant hyperchromatic epithelium, ranging from basaloid cells to large cells with vesicular nuclei [1,11].

DGCTs generally demonstrate lower proliferation indices compared to GCOC, particularly with the Ki-67 marker, although exceptions have been observed [12,13]. In GCOC, p53 expression varies widely, ranging from less than 25% to over 75%, while DGCT typically exhibits lower levels of p53 expression, with occasional reports of higher values. The lack of large-scale studies utilizing standardized methodologies has hindered the establishment of reliable cut-off values for these markers [14].

The treatment of DGCTs commonly includes enucleation, which involves the removal of the lesion while preserving the integrity of surrounding structures, or surgical resection, which entails excising the tumor with an adequate margin of healthy tissue to ensure complete removal. Surgical resection can include techniques such as segmental resection, where a portion of the bone containing the tumor is removed while maintaining jaw continuity, or en bloc removal, which excises the tumor along with surrounding bone or soft tissue as a single unit, depending on the aggressiveness of the lesion [15,16].

Prolonged follow-up is strongly recommended, as recurrence has been documented within five years following interventions such as segmental mandibular resection. Central DGCTs demonstrate aggressive and locally invasive characteristics, with reported recurrence rates reaching up to 71% [15,16]. In contrast, peripheral DGCTs display less aggressive behavior and are typically not linked to recurrence.

The primary treatment for GCOC is extensive surgical resection with at least a 5 mm margin, achieving success in approximately two-thirds of cases [10]. Adjuvant radiotherapy has been used in a minority of patients, although its benefits remain uncertain. For cases with lymph node metastasis, aggressive multimodal approaches and immunotherapy have been suggested.

## Conclusions

This case emphasizes the importance of accurate histopathological evaluation in distinguishing a DGCT from its malignant counterpart, GCOC, due to differences in prognosis and treatment strategies. The decision to perform enucleation over more extensive resection highlights the importance of tailoring management to the patient’s comorbidities and overall risk profile. Vigilant clinical and radiographic follow-ups are crucial, given the significant recurrence rates associated with central DGCTs. This report contributes to the limited literature on DGCTs, providing valuable insights into their diagnosis, management, and clinical outcomes.
